# Underrepresentation of Nurses in Croatian Graduate Nursing Programs: Implications for Academic Equity in Nursing Education

**DOI:** 10.1111/jan.70388

**Published:** 2025-11-17

**Authors:** Ivana Žilić, Sanda Franković, Hana Brborović, Marko Marelić, Ognjen Brborović

**Affiliations:** ^1^ Nursing Home Senior Garden Zagreb Croatia; ^2^ Ambitio College Zagreb Zagreb Croatia; ^3^ School of Medicine University of Zagreb Zagreb Croatia; ^4^ High School for Nurses “Mlinarska” Zagreb Croatia; ^5^ Environmental Health, Occupational and Sports Medicine Department University of Zagreb, School of Medicine Zagreb Croatia; ^6^ Medical Sociology and Health Economics Department University of Zagreb, School of Medicine Zagreb Croatia; ^7^ Social Medicine and Organization of Health Care Department University of Zagreb, School of Medicine Zagreb Croatia

**Keywords:** academic representation, faculty development, gender and nursing, graduate programs, higher education, nursing education, nursing leadership, nursing workforce, professional equity

## Abstract

**Aim:**

To examine the representation of nurses in Croatian graduate nursing programs and to explore its implications for academic equity, professional development, and nursing leadership.

**Design:**

Cross‐sectional descriptive study using document analysis.

**Methods:**

Data on lecturers and course leaders for the 2022/2023 academic year were retrieved from all graduate and specialist nursing programs in Croatia. A total of 694 lecturers and 545 course leaders were analysed by professional background.

**Results:**

Nurses accounted for 19% of lecturers in university graduate programs and 14% in specialist studies. As course leaders, they represented 11% in specialist studies and only 4% in university graduate programs. Thirty courses lacked assigned lecturers. The findings demonstrate a strong dominance of medicine and other professions in teaching roles.

**Conclusion:**

Nurses remain markedly underrepresented in academic positions, which may limit their ability to shape curricula, influence educational standards and strengthen professional identity.

**Implications for the Profession and/or Patient Care:**

Improving the academic presence of nurses could be important for advancing leadership capacity, curriculum relevance and professional equity. Stronger representation may help enhance the profession's authority and indirectly benefit patient care.

**Impact:**

*What problem did the study address?*: The study explored limited nurse representation in graduate nursing education and its implications for equity and autonomy. *What were the main findings?*: Nurses are a small minority in lecturer and course leader roles, with most positions occupied by non‐nursing professionals. *Where and on whom will the research have an impact?:* The findings are relevant to educators, regulators and policymakers in Croatia and internationally, particularly in countries where nurses face barriers to academic participation. The study supports ongoing policy efforts to strenghten nurses' academic representation and leadership in higher education.

**Patient or Public Contribution:**

No patient or public contribution. This study relied exclusively on publicly available academic data and did not involve patients, service users or members of the public.

## 1 Introduction

The distinction between an occupation and a profession is central to understanding nursing's academic position. While occupations involve specialised skills and knowledge, professions are defined by autonomy, ethical responsibility and control over training and standards (Professions Australia 2010). Sociological theories highlight that professional status depends on the ability of a group to regulate its own work and standards (Freidson 1970, 2001; Gabe et al. 2004). Evetts (2003) differentiates between occupational professionalism, marked by collegial authority, and organisational professionalism, characterised by external control, while Witz (1992) emphasises gendered closure strategies that have historically excluded women. These perspectives are important for nursing, a predominantly female profession shaped by medical dominance and systemic barriers.

Nursing has gradually transitioned from an apprenticeship model under physician supervision to a recognised academic discipline (Fealy and McNamara 2007; Mao et al. 2021). Early reforms, such as Nightingale's schools, reinforced gendered roles but also laid foundations for professional education. The development of nursing theories, including Henderson's, Neuman's and Roy's, supported the establishment of nursing as an academic discipline. In Europe, the Bologna Process harmonised nursing education, introducing a three‐cycle system and reinforcing competencies beyond clinical training, such as leadership and research (Collins and Hewer 2014; ten Hoeve et al. 2014; Laiho 2010). Despite this progress, nursing often continues to be perceived as low status and subordinate to medicine (Zhu et al. 2021; Piervisani et al. 2021; Esterhuizen and van Rensburg 2021; Pincha Baduge et al. 2023).

**FIGURE 1:**
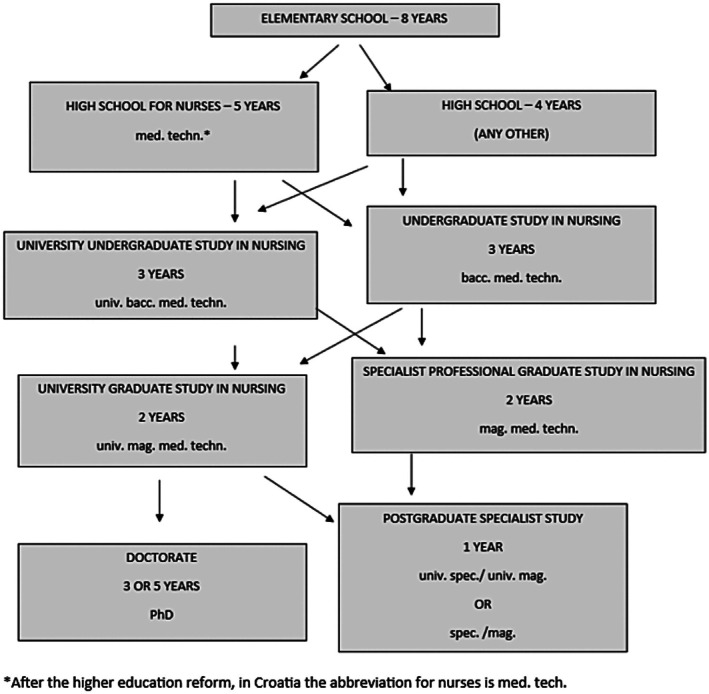
The trajectory of nursing education in Croatia. *Source:* Created by the authors.

In Croatia, the professionalrisation of nursing has advanced more recently. The first school for nurses was established in 1921 (Trgovec and Franković 2021) followed by the foundation of professional associations and regulatory frameworks, including the Croatian Chamber of Nurses and the Nursing Law in 2003 (Krajnović and Franković 2006; Franković 2021; Law on Nursing 2003). Specialist graduate programs began in 2006 and university graduate programs in 2010 (Mrnjec 2014). By 2022/2023, 16 master's programs were active across 10 faculties, establishing a complete educational vertical. Accreditation and quality assurance are overseen by the Agency for Science and Higher Education and the Ministry of Science and Education. The trajectory of nursing education in Croatia is shown in Figure 1.

Underrepresentation of nurses in academia is both a professional and an equity issue. In Croatia, 38% of the public still view nurses as physician's assistants (Neuberg et al. 2024), which undermines the recognition of nursing as an autonomous academic discipline. International comparisons show contrasts: in Ireland, the UK and the United States, nurses make up the majority of academic staff in nursing schools (McNamara 2008; School of Nursing and Midwifery 2024; Yale University 2024), while in Germany, Slovenia and neighbouring countries their role remains limited (University of Maribor 2024; University of Mostar 2024; Catholic University of Applied Health Sciences 2024). These patterns highlight how systemic, institutional and gendered dynamics influence academic participation.

This study therefore focuses not on professional autonomy in its broad sociological sense, but on a specific and measurable dimension of professional equity: the academic representation of nurses in Croatian graduate programs. This approach provides evidence on the extent to which nurses are positioned to influence curricula and educational standards, which are essential for the development of the profession.

This topic has broader international relevance, as the underrepresentation of nurses in academic roles has been identified in several European and global contexts, affecting professional development, equity and health workforce sustainability (OECD 2024; WHO 2020).

### 1.1 Research Questions and Hypotheses

To address this aim, the study was guided by two research questions:

What is the proportion of nurses in academic teaching roles compared to other professional groups in Croatian graduate nursing programs?

What are the potential implications of this representation for the profession's ability to shape education and sustain its academic development?

No formal hypotheses were tested, as the study employs a descriptive design. The emphasis is placed on documenting current representation and discussing its implications.

## 2 Materials and Methods

The aim of this study was to describe and explore the representation of nurses in academic roles within Croatian graduate nursing programs. Specifically, the study examined both university graduate programs and specialist professional graduate studies, focusing on the distribution of lecturers and course leaders by professional background. The study is descriptive and exploratory in nature, based on publicly available data, and does not test formal hypotheses. Ethical approval was obtained from the School of Medicine, University of Zagreb. No interviews were conducted, and no personal data were collected.

### 2.1 Study Design

A descriptive, cross‐sectional design was employed. The study included all graduate‐level nursing programs in Croatia, thereby providing comprehensive national coverage. Data were obtained from publicly accessible university sources, including study plans, course listings and faculty websites, collected between November 1, 2022, and September 29, 2023. This approach ensured transparency and allowed for an indicative comparison across institutions.

### 2.2 Inclusion Criteria

- Graduate‐level nursing programs in Croatia (university and specialist professional).
- Officially published data on faculty and courses for the 2022/2023 academic year.
- Information accessible through university websites and study programs.

Programs without graduate‐level nursing studies were excluded. Missing or incomplete data for some courses is acknowledged as a limitation of the study and is described in the Section 5 section.

### 2.3 Data Collection and Classification

For each program, all lecturers and course leaders were identified from official documents. In cases with multiple lecturers per course, each was counted once to avoid overrepresentation of individuals. Professional background was determined using:

- Academic title and designation.
- Highest degree (e.g., MSc in Nursing, MD, MSc in Psychology).
- Institutional affiliation when provided.

Lecturers were classified into three groups:

1. Nurses (holding degrees in nursing).
2. Physicians (MDs).
3. Other professions (e.g., psychologists, pedagogues, economists, sociologists, lawyers, physiotherapists and others).

This categorisation reflects the interdisciplinary nature of nursing education and allows comparison of contributions by different professional groups.

### 2.4 Data Analysis

Descriptive statistics were applied to calculate the number and percentage of lecturers in each category. Results were summarised in tables and figures to illustrate distributions across faculties and programs. Separate analyses were conducted for university graduate programs and specialist professional graduate studies to capture potential differences in staffing structures.

The analysis accounted for the possibility that some lecturers taught multiple courses. Everyone was counted once, based on their primary role, to avoid inflation of representation.

## 3 Results

**CHART 1:**
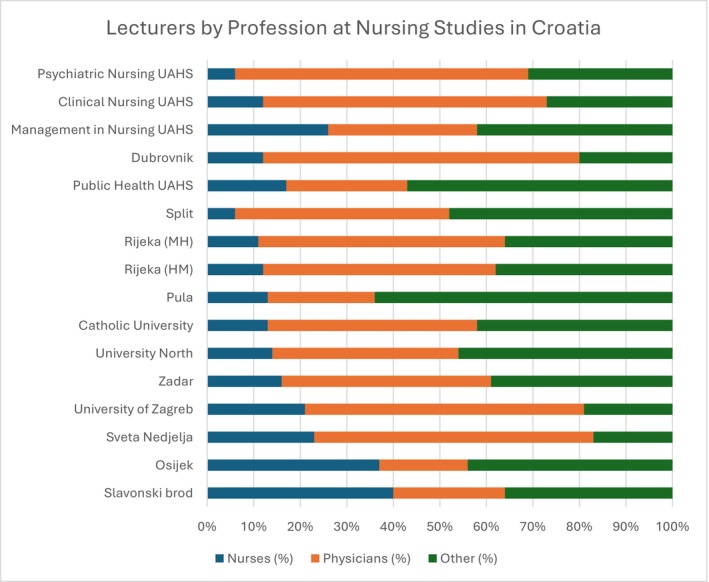
Lecturers by profession at nursing studies in Croatia. *Source:* Created by the authors.

Lecturers in Croatian graduate nursing studies come from three main professional groups: nurses, physicians and other professions. The category of “other professions” includes psychologists, educational rehabilitators, pedagogues, theologians, economists, teachers, journalists, sanitary engineers, physiotherapists, political scientists, sociologists, nutritionists, lawyers, librarians and biologists. This demonstrates the interdisciplinary nature of nursing education in Croatia (Table 1; Chart 1).

**TABLE 1:** Comparison of lecturers by profession at graduate nursing studies in Croatia.

Ordinal number	University^a^	Nurses *N* (%)	Physicians *N* (%)	Other *N* (%)
University studies				
1	Faculty of Dental Medicine and Health, Josip Juraj Strossmayer University of Osijek, dislocated study Slavonski Brod	13 (40%)	8 (24%)	12 (36%)
2	Faculty of Dental Medicine and Health, Josip Juraj Strossmayer University of Osijek	10 (37%)	5 (19%)	12 (44%)
3	Faculty of Dental Medicine and Health, Josip Juraj Strossmayer University of Osijek, dislocated study Sveta Nedjelja	11 (23%)	28 (60%)	8 (17%)
4	School of Medicine, University of Zagreb	28 (21%)	80 (60%)	26 (19%)
5	Department of Health Studies, University of Zadar	9 (16%)	25 (45%)	22 (39%)
6	University North	7 (14%)	19 (40%)	22 (46%)
7	Catholic University of Croatia	6 (13%)	21 (45%)	20 (42%)
8	Juraj Dobrila University in Pula	4 (13%)	7 (23%)	20 (64%)
9	Faculty of Health Studies, University of Rijeka, Healthcare Management	3 (12%)	12 (50%)	9 (38%)
10	Faculty of Health Studies, University of Rijeka, Promotion and Protection of Mental Health	3 (11%)	15 (53%)	10 (36%)
11	University Department of Health Studies, University of Split	3 (6%)	21 (46%)	22 (48%)
Specialist professional studies				
1	University of Applied Health Sciences, Public Health	4 (17%)	6 (26%)	13 (57%)
2	University of Dubrovnik	7 (12%)	38 (68%)	11 (20%)
3	University of Applied Health Sciences, Management in Nursing	6 (26%)	5 (32%)	8 (42%)
4	University of Applied Health Sciences, Clinical Nursing	7 (12%)	36 (61%)	16 (27%)
5	University of Applied Health Sciences in Zagreb, Psychiatric Nursing	1 (6%)	10 (63%)	5 (31%)

*Note:* *Source:* Created by the authors.

^a^ Some of the lecturers are present in more than one study program.

The University of Josip Juraj Strossmayer in Osijek had the highest proportion of nursing lecturers (40%), particularly in the dislocated study in Slavonski Brod. In contrast, the University of Split and the University of Applied Health Sciences in Zagreb, especially the graduate specialist study in Psychiatric Nursing, had the lowest proportion (6%). A total of 30 courses lacked listed lecturers or course leaders.

**CHART 2:**
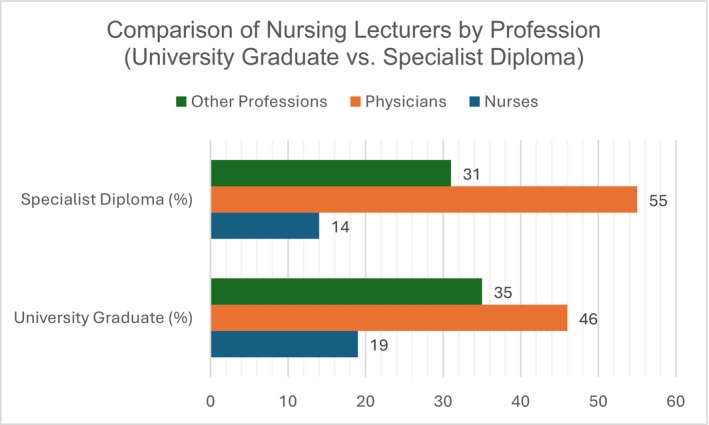
Comparison of nursing lecturers by profession (university graduate vs. specialist diploma). *Source:* Created by the authors.

In university graduate programs, a total of 521 lecturers were recorded: 241 (46%) physicians, 97 (19%) nurses and 183 (35%) from other professions. In specialist professional graduate studies, 173 lecturers were identified: 95 (55%) physicians, 25 (14%) nurses and 53 (31%) from other professions (Chart 2).

**CHART 3:**
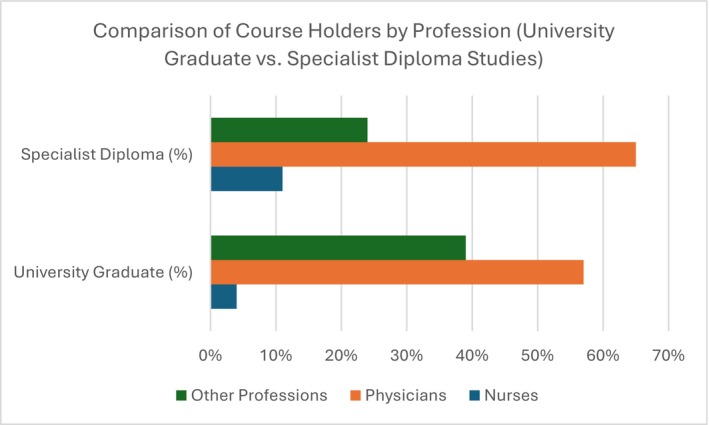
Comparison of course holders by profession (university graduate vs. specialist diploma studies). *Source:* Created by the authors.

Course holders showed similar trends. In specialist professional studies, 220 course holders were listed, with some individuals responsible for up to 13 courses. After adjusting for multiple teaching assignments, there were 116 unique course holders: 75 (65%) physicians, 13 (11%) nurses and 28 (24%) from other professions. In university graduate studies, 325 course holders were listed, with some teaching up to 12 courses. After adjustment, there were 198 unique course holders: 112 (57%) physicians, 8 (4%) nurses and 78 (39%) from other professions (Chart 3).

All analyses were based exclusively on publicly available faculty and curriculum records. The results provide a quantitative overview of professional distribution in Croatian graduate nursing education. These findings offer a descriptive snapshot of current academic staffing patterns and should be interpreted as indicative of broader trends rather than definitive causal relationships. Interpretation of these findings and their implications is addressed in the Section 4 section.

## 4 Discussion

This study suggests a marked underrepresentation of nurses in Croatian graduate nursing programs. Nurses comprised only 19% of lecturers in university graduate programs and 14% in specialist studies, while as course leaders they accounted for 11% and 4% respectively. In addition, 30 courses lacked assigned lecturers. These findings indicate potential disparities in academic staffing that may limit the profession's ability to influence curricula and educational standards (McNamara 2010).

The presence of nurse educators is widely recognised as crucial for professional development. By serving as mentors and role models, they strengthen students' professional identity and contribute to the autonomy of the discipline (Ryan and McAllister 2017; Ozdemir 2019; Leonard et al. 2010; McClunie‐Trust et al. 2024). Through the lens of Freidson's (1970) theory of professionalism, which emphasises self‐regulation as a defining feature of a profession, the limited representation of nurses in teaching roles may raise concerns about nursing's ability to fully govern its own education (Roshanzadeh et al. 2018; Rouhi‐Balasi et al. 2020). While professional autonomy is a broader sociological construct, academic representation is a measurable dimension of equity that directly affects educational self‐determination.

Several factors could help explain this underrepresentation. Gender dynamics remain a significant barrier. Nursing is a predominantly female profession, yet leadership roles in academic settings are often male‐dominated. Gender stereotypes appear to devalue women's work and may limit opportunities for women in academic leadership (Piervisani et al. 2021; Zhu et al. 2021; Shim and Park 2023). These dynamics likely hinder the progression of nurse educators to senior academic ranks (Pincha Baduge et al. 2023; Livesay et al. 2022; Bhatti and Ali 2021). Institutional structures also play a role (Perks 1993; McNamara 2009). In Croatia, many nursing programs are embedded within medical faculties, where appointments often prioritise physicians and allied health professionals, reducing opportunities for nurse faculty. In contrast, independent nursing faculties tend to employ more nurse academics.

In Croatia, even today in 2025, nurses do not have access to a standalone doctoral program in nursing; instead, they pursue doctoral education through related health sciences programs or abroad. By comparison, many Western countries had already established academic nursing decades earlier. Around 2010, fewer than 1% of nurses in Western Europe held a PhD (McNamara 2008), yet this still included hundreds of doctorally prepared nurses contributing to academia. EU directives on mutual recognition of qualifications (Piervisani et al. 2021) encouraged Croatia to align with European standards, leading to the introduction of graduate and specialist programs. Progress has been made, but challenges remain, including the absence of structured professional development pathways for academic careers, unlike medicine, which offers clear trajectories.

International comparisons further illustrate these differences. In Ireland, the UK and France, nurses constitute most faculty in nursing schools (School of Nursing and Midwifery 2024; City University of London 2024; University Outaouais and Laurentides 2024). In Germany, Bosnia and Herzegovina and Slovenia, nurses remain a minority in academia (Catholic University of Applied Health Sciences 2024; University of Mostar 2024; University of Maribor 2024). In North America, strong policies have ensured a robust academic nursing presence (Yale University 2024; University of Northern British Columbia 2024). In the U.S., recommendations to double the number of doctorally prepared nurses have shaped national strategies (Smeltzer et al. 2015).

In Croatia, however, there appears to be a pool of qualified nurses who could potentially strengthen academic representation. According to the Croatian Chamber of Nurses, there are 14 doctorally prepared nurses, five with a Master of Science degree (Mr. Sc.), and 2797 with a Master of Science in Nursing (M.Sc.). Despite this, institutional barriers may prevent their broader inclusion in teaching roles. This suggests that the challenge may not lie in a lack of qualified personnel but rather structural constraints within academic institutions.

Globally, a range of strategies has been used to strengthen nursing academia (Tsirigoti et al. 2024; Zappas et al. 2021). In the U.S., the Future of Nursing report (2010) called for 80% of RNs to hold a baccalaureate degree (McNamara 2008). In Europe, the Bologna Process and Directive 2005/36/EC standardised nursing qualifications, while the UK's Project 2000 integrated nursing into universities (Piervisani et al. 2021). Policy‐driven reforms, such as Ireland's government commission in the 1990s, transformed nursing education by moving all schools into universities by 2002 (Piervisani et al. 2021). Institutional investment has also proven influential.

Professional identity and leadership are also key dimensions. Nurses' identity develops through education and practice but is shaped by societal stereotypes of nursing as subordinate to medicine (Zou et al. 2024; Zhu et al. 2021). Leadership identity emerges through mentorship and academic engagement (Fitzgerald and Clukey 2021; Marcean and Alexandru 2022; Fischer 2017; Healey and Hays 2012). Yet, male nurses are often overrepresented in leadership positions due to stronger networks and mentorship (Santos et al. 2017). Overcoming these gendered patterns will likely require gender‐sensitive policies, leadership training and mentorship programs (Anicha et al. 2020; Ghamrawi et al. 2024; Wagner et al. 2012).

Taken together, these findings should be interpreted cautiously, as they are based on cross‐sectional, descriptive data. While the results indicate possible structural and gender‐related barriers, they do not establish causality. Future research incorporating qualitative or longitudinal methods may provide deeper insights into these complex relationships.

Overall, these findings suggest that while Croatia has aligned nursing education with European reforms, academic representation of nurses remains limited and should be viewed as part of a broader process of professional and institutional development. International evidence indicates that targeted policy frameworks, institutional investment and leadership development can help improve representation. Croatia may benefit from similar strategies, including funding for doctoral education, career pathways for nurse academics and gender equity policies. Addressing these barriers could strengthen nursing education and potentially enhance the profession's authority and contribution to healthcare.

## 5 Limitations and Future Research

This study has several limitations that should be acknowledged. First, the analysis relied exclusively on publicly accessible information from university websites and study plans, which may contain discrepancies or incomplete records. For example, 30 courses were listed without designated lecturers or course leaders. It is possible that these positions were filled but not published, which could have influenced the results. Similarly, some lecturers were responsible for multiple courses, but in the analysis each individual was counted only once to avoid overrepresentation.

Second, the data were collected between November 1, 2022, and September 29, 2023. The exclusion of course data from part of the 2022/2023 academic year introduces a temporal limitation, as more recent changes in staffing may not be reflected in the findings.

Third, the study provides a descriptive and exploratory overview of representation and should be interpreted accordingly. It does not investigate underlying causes or mechanisms that shape the observed patterns. Factors such as shortages of qualified nurse educators, administrative structures or institutional cultures were not explored. The analysis also does not consider differences between faculties, such as size, reputation or geographic location, which could influence academic staffing. In addition, the international comparison was based on a limited and unsystematic sample of countries, and therefore should not be generalised.

While this research highlights a low representation of nurses in academic roles, it does not establish a causal link between representation and educational outcomes. The findings should thus be viewed as indicative rather than conclusive, providing a basis for more detailed investigation.

Future research should address these limitations by incorporating qualitative interviews or focus groups with nurse educators and administrators, as well as systematic international comparisons. Longitudinal studies would allow changes in academic representation to be tracked over time. Given the gendered composition of the nursing workforce, future studies should also examine how gender equity intersects with access to academic careers and leadership opportunities.

## 6 Conclusions

This study suggests a significant underrepresentation of nurses in academic roles within Croatian graduate nursing programs. Nurses accounted for less than one‐fifth of lecturers and an even smaller share of course leaders, while physicians and other professionals dominated teaching positions. These disparities may limit the ability of nursing to contribute fully to the development of its own curricula and academic standards.

The findings indicate that intraprofessional education plays a crucial role in strengthening the nursing profession. Nurse educators provide more than subject expertise: they model professional identity, support student development and ensure that nursing education remains relevant to practice. However, these interpretations should be viewed as indicative rather than conclusive, given the descriptive nature of this research.

Policy and institutional actions could help address these imbalances. Universities and policymakers may consider prioritising the recruitment of nurse educators, creating structured mentorship and professional development pathways, and allocating resources for career progression in academia. Strengthening institutional support and ensuring transparent appointment processes could help overcome structural barriers to representation.

The underrepresentation of nurses must also be viewed through the lens of gender equity. As a predominantly female profession, nursing continues to face systemic barriers to leadership and authority in academia. Integrating gender‐sensitive strategies into academic workforce planning may therefore enhance both professional and gender equity. Tackling these inequities is not only essential for nursing education reform, but also for broader efforts to achieve Sustainable Development Goals on gender equality (SDG 5) and health system strengthening (SDG 3).

Overall, the study contributes context‐specific empirical evidence to international discussions on nursing education, academic equity and gender representation. Future policy reforms and institutional strategies should focus on building sustainable academic pathways for nurses, which would strengthen the profession, align Croatian nursing education with European and global standards, and potentially improve the quality and sustainability of healthcare.

## Author Contributions

Made substantial contributions to conception and design, or acquisition of data, or analysis and interpretation of data: I.Ž., S.F., O.B. Involved in drafting the manuscript or revising it critically for important intellectual content: I.Ž., S.F., H.B., M.M., O.B. Given final approval of the version to be published. Each author should have participated sufficiently in the work to take public responsibility for appropriate portions of the content: I.Ž., S.F., H.B., M.M., O.B. Agreed to be accountable for all aspects of the work in ensuring that questions related to the accuracy or integrity of any part of the work are appropriately investigated and resolved: I.Ž., S.F., H.B., M.M., O.B.

## Conflicts of Interest

The authors declare no conflicts of interest.

## Data Availability

The data supporting the findings of this study are available from publicly accessible official study sources and faculty websites. Due to the nature of this research, further data regarding specific course leaders and lecturers may be available upon reasonable request from the corresponding author.
